# A Cftr-independent, Ano1-rich seawater-adaptive ionocyte in sea lamprey gills

**DOI:** 10.1242/jeb.250110

**Published:** 2025-04-02

**Authors:** Ciaran A. Shaughnessy, Daniel J. Hall, Jessica L. Norstog, Andre Barany, Amy M. Regish, Diogo Ferreira-Martins, Jason P. Breves, Lisa M. Komoroske, Stephen D. McCormick

**Affiliations:** ^1^ Department of Integrative Biology, Oklahoma State University, Stillwater, OK 74078, USA; ^2^ Organismic and Evolutionary Biology, University of Massachusetts, Amherst, MA 01003, USA; ^3^ US Geological Survey, Eastern Ecological Science Center, Conte Research Laboratory, Turners Falls, MA 01376, USA; ^4^ Department of Biology, University of Massachusetts, Amherst, MA 01003, USA; ^5^ Departamento de Biología, Universidad de Cádiz, 11003 Cádiz, Spain; ^6^ Departamento de Genética, Fisiología y Microbiología, Universidad Complutense, 28040 Madrid, Spain; ^7^ Interdisciplinary Center of Marine and Environmental Research, University of Porto, 4450-208 Matosinhos, Portugal; ^8^ Department of Biology, Skidmore College, Saratoga Springs, NY 12866, USA; ^9^ Department of Environmental Conservation, University of Massachusetts, Amherst, MA 01003, USA

**Keywords:** Osmoregulation, Agnathan, Nka, Nkcc1, Evolution

## Abstract

All ionoregulating marine fishes examined to date utilize seawater-type ionocytes expressing the apical Cl^−^ channel, cystic fibrosis transmembrane conductance regulator (Cftr) to secrete Cl^−^. We performed transcriptomic, molecular and functional studies to identify Cl^−^ transporters in the seawater-type ionocytes of sea lamprey (*Petromyzon marinus*). Gill *cftr* expression was minimal or undetectable in larvae and post-metamorphic juveniles. We identified other Cl^−^ transporters highly expressed in the gills and/or upregulated following metamorphosis and further investigated two candidates that stood out in our analysis, a Ca^2+^-activated Cl^−^ channel, *anoctamin 1* (*ano1*), and the *Clc chloride channel family member 2* (*clcn2*). Of these, *ano1* was expressed 10–100 times more than *clcn2* in the gills; moreover, *ano1* was upregulated during seawater acclimation, while *clcn2* was not. Using an antibody raised against sea lamprey Ano1, we did not detect Ano1 in the gills of larvae, found elevated levels in juveniles and observed a 4-fold increase in juveniles after seawater acclimation. Ano1 was localized to seawater-type branchial ionocytes but, surprisingly, was localized to the basolateral membrane. *In vivo* pharmacological inhibition experiments demonstrated that a DIDS-sensitive mechanism was critical to the maintenance of osmoregulatory homeostasis in seawater- but not freshwater-acclimated sea lamprey. Taken together, our results provide evidence of a Cftr-independent mechanism for branchial Cl^−^ secretion in sea lamprey that leverages Ano1-expressing ionocytes. Once further characterized, the Cftr-independent, Ano1-rich ionocytes of sea lamprey could reveal novel strategies for branchial Cl^−^ secretion, whether by Ano1 or some other Cl^−^ transporter, not previously known in ionoregulating marine organisms.

## Teaser

**Summary:** Analysis of sea lamprey gills reveals a novel Cftr-independent, Ano1-rich ionocyte for salt secretion, suggesting greater diversity in marine vertebrate gill ionoregulatory mechanisms than previously known.

## INTRODUCTION

Lampreys and hagfishes are members of Class Agnatha, a sister taxon to all other vertebrates. Lampreys exhibit many morphological, physiological and genomic features that diverge from those of all other vertebrate groups. Thus, agnathans have been considered a keystone group in examining the origins and early evolution of various aspects of vertebrate biology (Xu et al., 2016). Lampreys, like most fishes, are ionoregulators, maintaining systemic ion (i.e. Na^+^ and Cl^−^) concentrations at different levels from their aquatic environment (Evans and Claiborne, 2008; Marshall and Grosell, 2006). Curiously, hagfishes appear to be the only exception to this pattern among fishes, tolerating internal Na^+^ and Cl^−^ concentrations that conform to those of the marine environment (Currie and Edwards, 2010).

Sea lamprey (*Petromyzon marinus*) are a euryhaline and anadromous species that migrate from freshwater (FW) to seawater (SW) as juveniles (Beamish, 1980). Before making their seaward migration, which can occur over a wide window spanning from autumn to spring (Shaughnessy and McCormick, 2022), anadromous lampreys undergo a months-long larvae-to-juvenile metamorphosis during the preceding summer and autumn. During this metamorphosis, the ability to survive in SW develops (Reis-Santos et al., 2008; Richards and Beamish, 1981; Shaughnessy and McCormick, 2020) as a result of preparatory increases in the hyposmoregulatory capacity of the gills and intestine (Barany et al., 2020, 2021a; Shaughnessy and McCormick, 2020; Reis-Santos et al., 2008), which is activated by endocrine factors (Barany et al., 2021b; Ferreira-Martins et al., 2023; Gong et al., 2020, 2022; Shaughnessy et al., 2020). This developmental preparation for SW entry in lampreys is strikingly similar to the widely studied parr–smolt transformation in salmonids (McCormick, 2013; Shaughnessy and Bystriansky, 2023).

The secretion of excess salts is key to the survival of ionoregulating fishes in SW. Salt-secreting epithelia in vertebrates exist in a variety of organs, including the elasmobranch rectal gland, marine fish gill, amphibian skin and reptilian lingual and avian nasal salt glands (Schlatter and Greger, 1988). These organs, although disparate and non-homologous, leverage a remarkably similar cellular strategy to achieve Cl^−^ secretion. The defining feature of the salt-secreting ionocyte in these organs is the co-expression of two basolateral ion transporters, Na^+^/K^+^-ATPase (Nka) and Na^+^/K^+^/2Cl^−^ cotransporter 1 (Nkcc1), along with an apical Cl^−^ channel, the cystic fibrosis transmembrane conductance regulator (Cftr) (Schlatter and Greger, 1988). In these ionocytes, Cftr is the primary conduit for Cl^−^ secretion. This Nka-, Nkcc1- and Cftr-rich ionocyte was first characterized in the shark rectal gland (Riordan et al., 1994) and later in the marine actinopterygian fish gill (Marshall and Singer, 2002; Marshall et al., 1995, 2002b; McCormick, 2003; Singer et al., 1998) as the key cellular pathway for Cl^−^ secretion that is essential to the maintenance of ionic homeostasis in marine environments. Although this molecular mechanism for Cl^−^ secretion has only been examined in a relatively few ionoregulating marine fishes, all species examined to date exhibit a Cftr-rich branchial ionocyte. Indeed, it has been widely presumed that all ionoregulating marine fishes, including marine lampreys, cartilaginous fishes and actinopterygians use a similar Nka-, Nkcc1- and Cftr-rich, Cl^−^-secretory ionocyte to ionoregulate in SW (Bartels and Potter, 2004; Evans and Claiborne, 2008; Evans et al., 2005; Hiroi and McCormick, 2012; Marshall and Grosell, 2006).

Our previous research has characterized the coupled roles of Nka and Nkcc1 in the SW-type branchial ionocyte of sea lamprey (Reis-Santos et al., 2008; Shaughnessy and McCormick, 2020). However, it remains unresolved whether Cftr facilitates apical Cl^−^ secretion in these branchial ionocytes (Ferreira-Martins et al., 2021; Shaughnessy and Breves, 2021). Here, we performed experiments during key developmental events in the sea lamprey life cycle, the larvae-to-juvenile metamorphosis and SW acclimation of juveniles. Then, we employed transcriptomic, molecular and functional analyses to identify and characterize candidate Cl^−^ channels expressed by SW-type ionocytes that may facilitate Cl^−^ secretion in lieu of Cftr. This investigation thus represents a step toward a more nuanced understanding of the diversity of ion transport mechanisms that exist in vertebrate SW-type ionocytes.

## MATERIALS AND METHODS

### Animal care and experiments

All live animal experimentation was performed at the Conte Anadromous Fish Research Center (US Geological Survey, Turners Falls, MA, USA) with approval by and in accordance with the Institutional Animal Care and Use Committees at the University of Massachusetts at Amherst (Protocol No. 2016-0009) and the US Geological Survey (Protocol No. SP9065). For the metamorphic series, sea lamprey, *Petromyzon marinus* Linnaeus 1758, were collected monthly from the wild (Connecticut River watershed, Montague, MA, USA) throughout the natural course of metamorphosis in a single year (2016, June to November), then either sampled immediately upon capture (metamorphic profile experiment) or transported to the laboratory for further experimentation (salinity experiments). In the laboratory, salinity experiments with juvenile sea lamprey were carried out under natural photoperiod at 15°C in recirculating 1.5 m diameter fiberglass tanks (salinity time-course experiment) or 60 l glass aquaria (tissue profile and pharmacological inhibition experiments) supplied with filtered and dechlorinated municipal water (FW). For salinity experiments, 35‰ water (SW) was prepared by dissolving an artificial sea salt formula (Crystal Sea Salt, Baltimore, MD, USA) into dechlorinated municipal water.

For our pharmacological inhibition experiments, we investigated whether Cftr was necessary for osmoregulation in SW by exploiting previously established pharmacological approaches for investigating Cl^−^ channel function in fishes. DIDS (4,4′-diisothiocyano-2,2′-stilbenedisulfonic acid, Sigma) is a Cl^−^-channel blocker known to inhibit the activity of many anion transporter types (Harding et al., 2024). However, DIDS does not block Cftr function (Pedemonte and Galietta, 2014; Schultz et al., 1999). The insensitivity of Cftr to DIDS has been utilized to characterize Cftr-specific functions in teleost ionoregulatory epithelia (Marshall et al., 2002a). Here, DIDS was dissolved in dimethyl sulfoxide and added to either FW or SW for a final concentration of 50 µmol l^−1^ (aqueous exposure) or added to saline for a final administration dose of 1 μmol g^−1^ body mass (intraperitoneal injection). Then, FW-acclimated juvenile sea lamprey were exposed to either FW or SW with DIDS or a vehicle control for 24 h prior to plasma collection. Determination of these doses was based on previous work with DIDS on teleost tissue *in vitro* (Marshall et al., 1995, 2002a) as well as our own experience with aqueous and intraperitoneal drug administration in sea lamprey (Shaughnessy et al., 2020; Shaughnessy and McCormick, 2020). From our approximation, the extracellular concentration of DIDS resulting from intraperitoneal injection should be similar to the concentration used during aqueous exposure.

Prior to sampling, sea lamprey were euthanized using a lethal dose of MS-222 (200 mg l^−1^) buffered with NaHCO_3_ (pH 7.0). Blood was collected from lamprey caudal vasculature using capillary tubes and then centrifuged for plasma separation. Blood plasma and tissues were frozen and stored at −80°C.

### Transcriptomic analyses

Total RNA was isolated from frozen gill tissue from larval and juvenile sea lamprey using TRI Reagent (Molecular Research Center Inc., Cincinnati, OH, USA). The concentration and purity of each extracted RNA sample were spectrophotometrically determined using a Take3 microvolume plate (BioTek Instruments, Winooski, VT, USA). The integrity of each extracted RNA sample was assessed via gel electrophoresis. Only RNA samples with high purity (1.9<*A*_260_/*A*_280_<2.2) and high integrity (determined by visually assessing band intensities of 28S and 18S rRNA) were used for transcriptomic analysis. Sequencing libraries were generated using the NEBNext Ultra Directional RNA Library Prep Kit for Illumina and the NEBNext Multiples Oligos for Illumina (New England, Biolabs, Ipswich, MA, USA) for dual indexing with modifications for half reactions. Pooled libraries (equimolar quantities) were sequenced by running 150 bp paired-end reads on an Illumina NextSeq 500 using a NextSeq500 Mid Output kit (Illumina) at the University of Massachusetts Amherst Genomics Resource Laboratory. The quality of unprocessed sequencing reads was assessed using FastQC (http://www.bioinformatics.babraham.ac.uk/projects/fastqc/, accessed August 2022). Pre-alignment removal of adaptor sequences and low-quality reads were carried out using Sickle (v1.33) (https://github.com/najoshi/sickle). This filtering resulted in an average of 44.38 million reads (22.19 million pairs) kept and 15.59 million reads (7.80 million pairs) discarded per sample. Trimmed, paired-end reads were mapped (100% map rate) to the sea lamprey reference genome (GenBank: GCA_010993605.1) using STAR Aligner (v2.7.9a) (Dobin et al., 2013). Gene-level counts (fragments per million) were calculated using DESeq2 with robust normalization (v1.32.0) (Love et al., 2014). This list of differentially expressed genes was screened for genes encoding Cl^−^ transporters that could plausibly have roles in branchial Cl^−^ transport (an *a posteriori* selection). Additionally, the entire transcriptome was screened for genes encoding Cl^−^ channels that were suspected *a priori* to have a role in branchial ionoregulation (e.g. *cftr*). The complete *a priori* and *a posteriori* selection of genes (Table S1) was analyzed.

### Quantitative real-time PCR

Total RNA was extracted from frozen tissue using TRI Reagent and spectrophotometrically analyzed for quantity and purity using a Take3 microvolume plate. First-strand cDNA synthesis was performed using the High-Capacity cDNA Reverse Transcription Kit (Life Technologies, Carlsbad, CA, USA). Quantitative real-time PCR (qPCR) was carried out with 2 ng cDNA, 200 nmol l^−1^ forward and reverse primers and SYBRSelect master mix (ThermoFisher Scientific, Waltham, MA, USA) in 10 µl reactions using the following protocol: activation at 95°C for 2 min, then 40 cycles of 95°C for 15 s, 60°C for 1 min, then 72°C for 30 s. A single product was confirmed by melt curve analysis and electrophoresis. The reference gene *ef1a* served as an internal control. Primer pairs for sea lamprey *nka*, *nkcc1* and *ef1a* were identical to those previously published (Shaughnessy and McCormick, 2020). Primer sequences for sea lamprey *cftr* were: forward, 5′-CTGAAAGGGCTGTGGACGAT-3′; reverse, 5′-CAGGGCTTGGTGGAAGATGT-3′. Primer sequences for sea lamprey *anoctamin 1* (*ano1*) were: forward, 5′-AAGTACAGCATGGGCGTCAA-3′; reverse, 5′-ACTGTGCAGCAGCTTACGAT-3′. Primer sequences for sea lamprey *chloride ion channel 2* (*clcn2*) were: forward, 5′-GAAGAGATAGAGCGGTGGGC-3′; reverse, 5′-TCCTGCGTGTACCTCCCATA-3′. The comparative 2^−ΔΔCt^ method (Pfaffl, 2001) was used to calculate relative mRNA transcript abundance. To compare relative mRNA transcript abundance of different sea lamprey genes within samples, 2^−ΔCt^ values were used. For visual clarity, relative mRNA transcript abundance was then presented with the lowest abundance transcript (*cftr*) set to 1 on a log scale.

### Antibodies

Nka was detected using the ‘α5’ mouse-monoclonal antibody (AB_2166869) and Nkcc1 was detected using the ‘T9’ mouse-monoclonal antibody (AB_2618107), both obtained from the Developmental Studies Hybridoma Bank (Iowa City, IA, USA). The use of these antibodies in sea lamprey has been previously described (Reis-Santos et al., 2008; Shaughnessy and McCormick, 2020).

To immunodetect Ano1, a custom-made rabbit-polyclonal antibody was raised against a region at the N-terminal side (Ala199–Glu498) of sea lamprey Ano1 (GenBank accession no. XP_032833934.1), which includes intracellular, transmembrane and extracellular domains. This anti-sea lamprey Ano1 antibody was produced by Boster Bio (Pleasanton, CA, USA). The specificity and affinity of the sea lamprey Ano1 antibody to the purified recombinant sea lamprey Ano1 peptide antigen was confirmed via an enzyme-linked immunosorbent assay provided by the manufacturer. Western blotting confirmed that the antibody produced a single band at the expected molecular weight (∼125 kDa) of sea lamprey Ano1 (Fig. S1). We also confirmed via immunofluorescence microscopy that the antibody did not show non-specific binding in cell types other than mature SW-type ionocytes. Additionally, the sea lamprey Ano1 antibody was suitable for use in Western blotting given that no non-specific binding of the secondary antibody was observed in a blot containing gill proteins and incubated without the Ano1 antibody. Moreover, a linear relationship was observed between antibody-specific band intensity and the amount of gill protein sample loaded into each lane (0.2 to 20 μg per lane). For immunofluorescence microscopy, a negative control incubated with only the secondary antibody further confirmed the specificity of the Ano1 antibody.

Additionally, in our immunofluorescence microscopy analyses, we probed for Cftr signal in the gills of SW-acclimated sea lamprey using the ‘24-1’ mouse-monoclonal antibody (AB_2260673; R&D Systems, Minneapolis, MN, USA), which was raised against the C-terminal region (residues 1377–1480) of the human CFTR. This antibody has been used successfully to detect Cftr in the gills of marine teleosts (Marshall et al., 2002b; McCormick, 2003).

### Western blotting

Gill tissue was homogenized in SEID buffer (150 mmol l^−1^ sucrose, 10 mmol l^−1^ EDTA, 50 mmol l^−1^ imidazole and 0.1% sodium deoxycholate, pH 7.3) containing an EDTA-free protease inhibitor cocktail (Pierce, Rockford, IL, USA). The homogenate was then centrifuged at 2000 ***g*** for 5 min. The crude supernatant was used for protein detection and quantification of abundance by western blotting. Protein content was determined using the BCA Protein Assay (Thermo Scientific, Rockford, IL, USA) and the samples were diluted in Laemmli sample buffer to an equal concentration, denatured by heating for 15 min at 60°C, and stored at −80°C. Samples (10 µg protein per lane along with a protein standard) were electrophoretically separated on a 7.5% SDS-PAGE (sodium dodecyl sulfate polyacrylamide gel electrophoresis) mini-gel (Bio-Rad, Hercules, CA, USA), then electrophoretically transferred to a polyvinylidene fluoride (PVDF) membrane (Millipore, Bedford, MA, USA). After transfer, PVDF membranes were equilibrated to phosphate-buffered saline containing 0.05% Triton X-100 (PBS-Tx), blocked for 1 h at 23°C in 5% non-fat milk in PBS-Tx, then incubated overnight at 4°C in a 1:2000 dilution of primary antibody (final concentration of antibody was 0.25 µg ml^−1^). After primary incubation, membranes were washed in PBS-Tx, then incubated for 2 h at 23°C in a 1:10,000 dilution of a horseradish peroxidase (HRP)-conjugated goat anti-mouse secondary antibody (Kirkegaard & Perry Laboratories, Gaithersburg, MA, USA) in blocking buffer. After secondary antibody incubation, membranes were washed again with PBS-Tx. Antibody-bound Ano1 protein was detected via enhanced chemiluminescence (ECL) using a homemade ECL solution, a 1:1 mixture of solution A (396 μmol l^−1^ coumaric acid, 2.5 mmol l^−1^ luminol, 100 mmol l^−1^ Tris-HCl, pH 8.5) and solution B (0.018% H_2_O_2_, 100 mmol l^−1^ Tris-HCl, pH 8.5). Chemiluminescence was imaged by a Syngene PXi system (SYNGENE Inc., Frederick, MD, USA). Band intensity was measured using ImageJ (National Institutes of Health, Bethesda, MD, USA). Protein was quantified relative to the average signal detected in the FW juvenile gill samples, which was set to a value of 1.

### Immunofluorescence microscopy

Whole gill pouches from six individual sea lamprey were placed in 4% paraformaldehyde (PFA) in 10 mmol l^−1^ PBS at room temperature. Gill tissue was kept in 4% PFA solution for 2 h to ensure complete fixation, then rinsed with and preserved in 70% ethanol at 4°C. In preparation for sectioning, gill tissue was rinsed in PBS and equilibrated to 30% (w/v) sucrose in PBS. Prepared gill tissue was then embedded in paraffin. Embedded gill tissue was sectioned (5 μm thickness) and mounted on microscope slides (FisherBrand Colorfrost Plus, Fisher Scientific, Hampton, NH, USA). Tissue sections were deparaffinized by immersing slides in fresh xylene for 5 min twice, then rehydrated through a graded ethanol series, followed by a final rinse in distilled water, and equilibration with PBS before proceeding with blocking and primary antibody incubation. Before incubation with primary antibody, tissue sections were incubated for 30 min in blocking solution (10% normal goat serum in PBS). Tissue sections were then incubated with anti-Nka α5 antibody (1:1000 dilution) or anti-Ano1 antibody (1:500; 1 µg ml^−1^) overnight at 4°C in antibody dilution buffer (0.01% NaN_3_, 0.1% BSA and 2% normal goat serum in PBS). After primary incubation, slides were washed in PBS and then incubated for 2 h at room temperature in fluorescent-labeled secondary antibody (goat anti-mouse or goat anti-rabbit) diluted 1:1000 in antibody dilution buffer. After secondary incubation, slides were again washed in PBS, covered with a coverslip, and examined with an inverted fluorescence microscope (20×) with a mercury lamp (Zeiss, Hebron, KY, USA).

### Calculations and statistical analyses

Group data are presented as means±s.e.m. Statistical comparisons between groups were assessed using Student's *t*-test, or one-way or two-way ANOVA with Tukey's *post hoc* test. All *n* values are presented in figure legends. Assumptions of normality and heterogeneity of variance were confirmed by Shapiro–Wilk and Brown–Forsythe tests, respectively. For all statistical tests, α=0.05. GraphPad Prism 6.0 (La Jolla, CA, USA) was used for all statistical analyses and figure preparation.

## RESULTS

### Transcriptomic analysis of Cl^−^ channels in sea lamprey gills

**Fig. 1.**
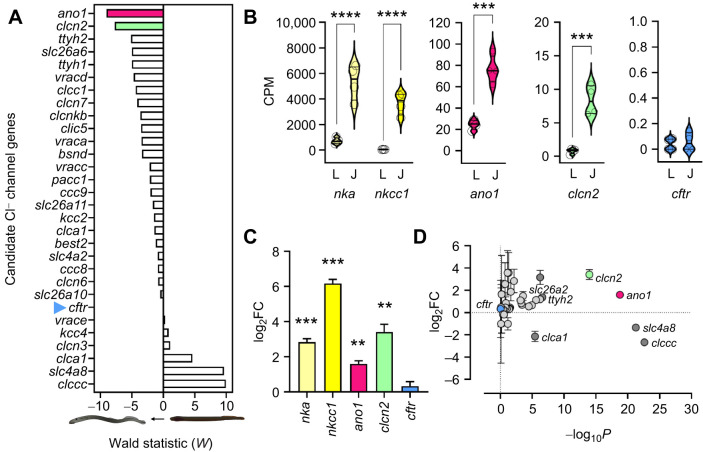
**Transcriptomic analysis of Cl^−^ channels in the gills of sea lamprey.** (A) Wald test analysis for a combined *a priori* and *a posteriori* selection of plausible apical Cl^−^ transporters from RNAseq analysis of larvae and juvenile lamprey gills. Values that are more negative indicate more highly expressed and/or differentially upregulated transcripts in the gills of juvenile sea lamprey (*n*=4). (B) Raw transcript abundance (counts per million, CPM) of *nka*, *nkcc1*, *ano1*, *clcn2* and *cftr* (two-sample *t*-test) in the gills of larvae (L) and juvenile (J) sea lamprey (*n*=4). (C) Fold-change (FC) in gill transcript abundance from larvae to juvenile of ion transporters (one-sample *t*-test against a reference value of 0) (*n*=4). (D) Volcano plot of Cl^−^ transporter transcript abundance, showing the magnitude of the fold-change (points above the log_2_FC=0 line are upregulated following metamorphosis; below this line they are down-regulated) and the strength of the significance (greater values for −log_10_*P* indicate greater significance). Asterisks denote significance (**P<0.01, ***P<0.001, ****P<0.0001).

Using next-generation RNA sequencing analysis guided by a newly released and updated assembly of the sea lamprey genome (Smith et al., 2018), we analyzed the branchial transcriptomes of larvae and juvenile sea lamprey for highly and differentially expressed ion transporters during metamorphosis (Fig. 1; Table S1). From this, we identified highly abundant and differentially expressed *nka* and *nkcc1* transcripts. It is notable that among a comprehensive list of *a priori*- and *a posteriori*-identified Cl^−^ channels with plausible roles in ionoregulation (Fig. 1A; Table S1), *cftr* was not highly or differentially expressed (Fig. 1). However, this approach revealed two other candidate Cl^−^ channels, *anoctamin 1* (*ano1*) and *chloride ion channel 2* (*clcn2*), that stood out from other Cl^−^ channels based on their high abundance in the gill and/or significant upregulation following metamorphosis (Fig. 1).

### Regulation of ion transporter expression across sea lamprey tissues and in the gills during metamorphosis and SW acclimation

**Fig. 2.**
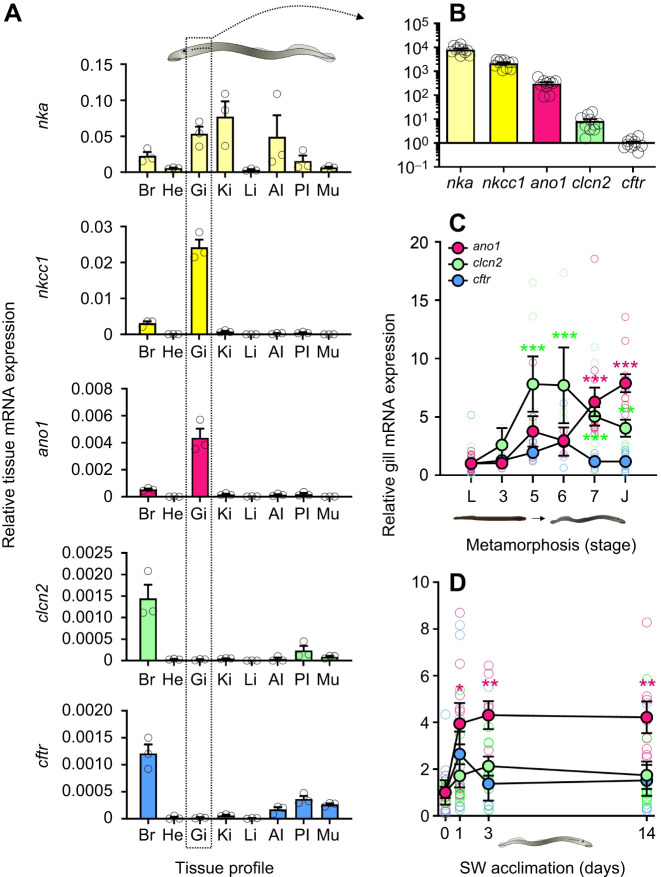
**Transcriptional regulation of *ano1*, *clcn2* and *cftr* in the gills of sea lamprey.** (A) Relative *nka*, *nkcc1*, *ano1* and *cftr* expression across various tissues in seawater (SW)-acclimated juvenile sea lamprey (*n*=3). Br, brain; He, heart; Gi, gill; Ki, kidney; Li, liver; AI, anterior intestine; PI; posterior intestine; Mu, muscle. (B) Relative transcript abundance of ion transporters in the gills of juvenile sea lamprey analyzed by qPCR (relative to *cftr*, which was set to a value of 1 for each individual) (*n*=10). (C,D) Relative gill *ano1*, *clcn2* and *cftr* expression during the larvae (L)-to-juvenile (J) metamorphosis (*n*=2–19) (C) and SW acclimation (*n*=3) (D). Asterisks indicate significant differences from larvae (C) or day 0 (D) (one-way ANOVA), which were set to 1 for each gene (*P<0.05, **P<0.01, ***P<0.001).

In a tissue profile of SW-acclimated juvenile sea lamprey, we observed high *nka*, *nkcc1* and *ano1* expression in the gills relative to other tissues (Fig. 2A). The kidney and anterior intestine also highly expressed *nka*. *nkcc1* expression was substantially higher (by at least 9-fold) in the gill compared with other tissues (Fig. 2A). Like *nkcc1*, *ano1* expression in SW-acclimated juveniles was substantially higher (9-fold) in the gill than in any other examined tissue (Fig. 2A). The highest levels of *clcn2* and *cftr* were found in the brain and posterior intestine (Fig. 2A). A direct comparison of the relative mRNA expression of these ion transporters in the sea lamprey gills revealed a hierarchy of expression: *nka=nkcc1*>*ano1*>>*clcn2*>>*cftr* (Fig. 2B).

We then compared the branchial expression of *ano1*, *clcn2* and *cftr* across various stages of metamorphosis and at multiple time points following exposure to SW. Branchial *clcn2* expression rose significantly (9-fold) during mid-metamorphosis (stages 5 and 6), then decreased slightly, although remaining significantly upregulated in juveniles (5-fold) (Fig. 2C). Branchial *ano1* expression rose significantly throughout metamorphosis, reaching its highest level of expression (9-fold) at the juvenile stage (Fig. 2C). Branchial *cftr* expression remained unchanged (*P=*0.541) throughout metamorphosis (Fig. 2C). After exposure of post-metamorphic juveniles to SW, branchial *ano1* expression increased by over 4-fold within 1 day and remained upregulated in SW (*P=*0.004) (Fig. 2D). Branchial *clcn2* and *cftr* expression did not significantly change upon SW exposure (*clcn2*, *P=*0.508; *cftr*, *P*=0.171) (Fig. 2D).

### Ano1 protein expression in sea lamprey gills

**Fig. 3.**
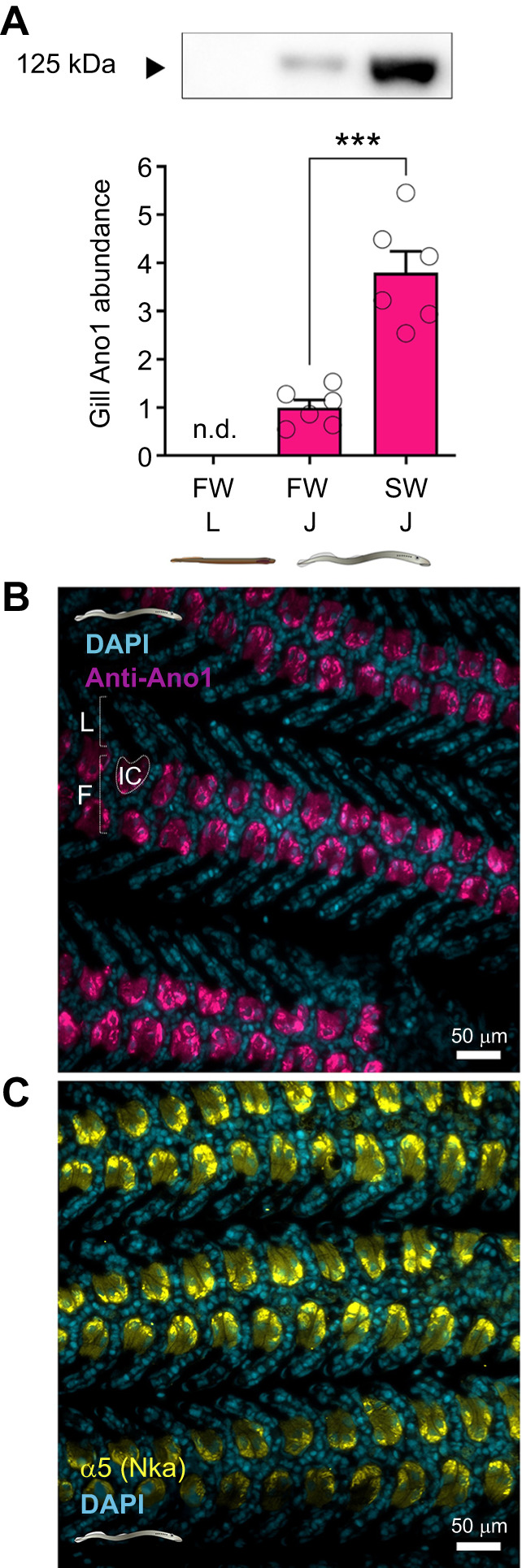
**Protein expression and cellular localization of Ano1 in gills of sea lamprey.** (A) Western blot comparison of Ano1 protein abundance in freshwater (FW)-acclimated sea lamprey before and after metamorphosis and seawater (SW) acclimation (L, larvae; J, juveniles) (*t*-test; *n*=6; ***P<0.001). Ano1 was not detected (n.d.) in larval gills. (B,C) Localization of Ano1 (magenta; B) and Nka (yellow; C) in gills of SW-acclimated juvenile sea lamprey. Ionocyte clusters (IC) are located along the filament (F) between lamellae (L).

We observed that Ano1 protein abundance was low (below detectable limits) in the gills of larvae and increased to detectable levels after metamorphosis (Fig. 3A; Fig. S1), reflecting the pattern of transcript-level expression of *ano1*. Ano1 protein abundance was almost 4-fold greater in the gills of SW- versus FW-acclimated juveniles (Fig. 3A), again reflecting patterns observed at the transcript level. Using immunofluorescence microscopy, secondary antibody-only controls produced no signal (Fig. S2), and we did not detect the presence of CFTR using the 24-1 antibody (Fig. S3). Ano1 was localized to Nka- and Nkcc1-rich SW-type ionocytes in the interlamellar spaces along the filament in SW-acclimated juvenile sea lamprey (Fig. 3B,C). Ano1 co-localized with Nka in the ionocytes clustered along the filament between the lamellae and appeared to primarily have a basolateral localization similar to that of Nka (Fig. 3B).

### Pharmacological inhibition of Ano1 reduces SW tolerance

**Fig. 4.**
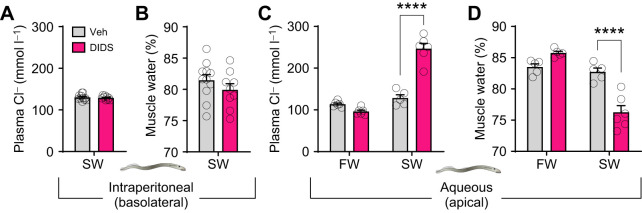
**Pharmacological disruption of osmoregulatory balance in seawater.** Plasma Cl^−^ concentration and muscle water content after intraperitoneal (*n=*5–6; A,B) or aqueous (*n=*9–12; C,D) exposure of freshwater (FW)- or seawater (SW)-acclimated juvenile sea lamprey to the Cl^−^ channel inhibitor 4,4′-diisothiocyano-2,2′-stilbenedisulfonic acid (DIDS; intraperitoneal, 1 μmol g^−1^; aqueous, 50 µmol l^−1^), a compound to which Cftr is insensitive. Veh, vehicle control. Data are means±s.e.m. (*n*=5–10). Asterisks denote significance of *t*-test (A,B) or two-way ANOVA (C,D) (****P<0.0001).

As Cftr is known to be insensitive to the broadly acting Cl^−^ channel blocker DIDS, we investigated whether osmoregulatory performance in SW was affected by exposure to DIDS. In SW-acclimated juveniles, intraperitoneal administration of DIDS had no effect (*P*=0.683) on plasma Cl^−^ levels, which remained at ∼120 mmol l^−1^ (Fig. 4A). Intraperitoneal administration of DIDS to SW-acclimated juveniles did not significantly affect muscle water content (*P*=0.268), which remained at ∼80% (Fig. 4B). Aqueous exposure to DIDS did not change (*P*=0.226) plasma Cl^−^ levels in FW-acclimated juveniles but did significantly increase (*P*<0.001) plasma Cl^−^ levels in SW-acclimated juveniles to ∼250 mmol l^−1^ (Fig. 4C). A similar effect of aqueous exposure to DIDS was observed for muscle water content; there was no effect of DIDS in FW-acclimated juveniles (*P=*0.107), whereas treatment of SW-acclimated juveniles with DIDS significantly decreased (*P*<0.001) muscle water content to ∼75% (Fig. 4D).

## DISCUSSION

Investigations into the histological, functional and molecular features of the sea lamprey branchial epithelium date back decades. Early investigations described that, during metamorphosis, larvae-specific mitochondria-rich cells (now referred to as FW-type ionocytes) on gill lamellae disappear and chloride cells (now referred to as SW-type ionocytes) appear in the interlamellar spaces of the filament (Peek and Youson, 1979). These SW-type ionocytes share many characteristics with those of marine teleosts (Bartels and Potter, 2004): (i) both have extensive membranous microtubule networks that expand the surface area of the basolateral membrane; (ii) both are rich with mitochondria; (iii) both form multicellular complexes, associating with adjacent cells to form ‘leaky’ paracellular junctions, presumably for the passage of Na^+^; and (iv) upon acclimation to SW, pavement cells covering the SW-type ionocytes recede, exposing the SW-type ionocytes to a greater share of the apical surface of the branchial epithelium. In contrast to the teleost SW-type ionocyte, sea lamprey ionocytes are arranged in groups along the filament, form paracellular junctions primarily with other ionocytes rather than accessory cells, and lack an apical crypt (Bartels and Potter, 2004).

It has generally been thought that the molecular mechanisms supporting Cl^−^ secretion by sea lamprey SW-type ionocytes are the same as those operating in the ionocytes of marine teleosts (Bartels and Potter, 2004). Indeed, our recent studies confirmed that some of the molecular components of sea lamprey SW-type ionocytes are similar to those found in teleosts. For example, large increases in Nka activity and abundance occur in sea lamprey gills during metamorphosis, and Nka is abundantly expressed in SW-type ionocytes on the filament (Reis-Santos et al., 2008). More recently, we co-localized Nkcc1 to the SW-type ionocyte and demonstrated that, like Nka, Nkcc1 abundance in the gills increases substantially during metamorphosis and after exposure to SW (Shaughnessy and McCormick, 2020).

The transcriptomic approach we employed here was in response to our initial efforts to investigate *cftr* in the sea lamprey gills. Here, we sought to broadly examine the transcriptomic changes in the gills following metamorphosis, when the molecular mechanisms of SW-type ionocytes are established (Reis-Santos et al., 2008; Shaughnessy and McCormick, 2020). We aimed to specifically identify which Cl^−^ transporter(s) may be involved in branchial Cl^−^ secretion in SW. Several types of Cl^−^ transporters were identified in our analysis as being abundant and/or differentially expressed in the gills during metamorphosis. It is remarkable that *cftr* shows minimal expression in the gills and is not upregulated following metamorphosis or SW exposure. If Cftr is indeed not supporting ionoregulation in sea lamprey gills, as our results indicate, then there are two possible explanations: (i) the gills of SW-acclimated sea lamprey do not secrete Cl^−^, or (ii) a novel molecular mechanism for Cl^−^ secretion is present in sea lamprey gills. Given the presence and abundance of the Nka- and Nkcc1-rich ionocytes in the juvenile and SW-acclimated sea lamprey gills, it appears more likely that a Cftr-independent mechanism for branchial Cl^−^ secretion exists within these cells.

Our transcriptomic approach to identifying this novel molecular mechanism for branchial Cl^−^ secretion in the sea lamprey gills is validated by the detection of upregulated *nka* and *nkcc1* transcription, reproducing our previous findings regarding the regulation of these ion transporters during metamorphosis (Shaughnessy and McCormick, 2020). Additionally, we identified several other Cl^−^ transporters that were differentially upregulated in the sea lamprey gills following metamorphosis. Among these, the differential expression of *ano1* and *clcn2* stood out. Other Cl^−^ transporters had either lower expression or weaker differential expression than *ano1* and *clcn2* and were not pursued further. It should be underscored that the gill transcript abundance and fold-changes following metamorphosis of *ano1* and *clcn2* are markedly lower than those of *nka* and *nkcc1*. For comparison, in Atlantic salmon (*Salmo salar*), branchial *cftr1* expression increases as much as, or more than, *nka* and *nkcc1* expression during the parr–smolt transformation or SW acclimation (Breves et al., 2017; Nilsen et al., 2007; Singer et al., 2002). The lack of a *clcn2* response to SW acclimation, and the robust upregulation of Ano1 transcript and protein expression during SW acclimation, led us to further investigate a potential role for Ano1. In fact, the 4-fold upregulation of Ano1 in the gills during SW acclimation is greater than the <2-fold increase in expression we observed for Nka and Nkcc1 (Shaughnessy and McCormick, 2020). Therefore, the mechanism for Cl^−^ secretion in the sea lamprey gills might be fully established only upon SW exposure, rather than during metamorphosis, as appears to be the case for Nka and Nkcc1.

Our finding that Ano1 is highly expressed in the Nka- and Nkcc1-rich ionocytes in sea lamprey gills is, to our knowledge, the first instance of Ano1 protein being localized to the SW-type ionocyte of fishes. Ano1 is functionally similar to Cftr in many ways (Jang and Oh, 2014): (i) Ano1 and Cftr are both apical Cl^−^ channels expressed in salt-secreting epithelia, (ii) Ano1 and Cftr are both able to facilitate the transport of bicarbonate, and (iii) Ano1 and Cftr are known to couple with Nkcc1 in salt-secreting cell types. Unlike Cftr, which is phosphorylation-activated by a cAMP/protein kinase A pathway, Ano1 is activated by the direct binding of Ca^2+^/calmodulin complexes. Both pathways of activation allow for rapid modulation of Cl^−^ secretion. Although our immunofluorescence microscopy results clearly localize Ano1 to the clusters of Nka-rich SW-type ionocytes in the sea lamprey gill, further microscopic analyses will be needed to determine the precise localization of Ano1 to help resolve its role in these ionocytes.

Other recent work in teleosts has similarly identified a potential role for Ano1 in osmoregulation in SW. In three-spine stickleback (*Gasterosteus aculeatus*), transcriptional expression of *ano1* is higher in the gills of the SW ecotype than in the FW ecotype (Taugbøl et al., 2022). In Atlantic killifish (*Fundulus heteroclitus*), branchial *ano1* expression is upregulated upon SW exposure and downregulated upon FW exposure (Breves et al., 2024; Tao and Breves, 2024), and further downregulated by prolactin, a FW-adaptive hormone (Breves et al., 2024). In the gills of Japanese medaka, *ano1* was upregulated during exposure to SW and after treatment with cortisol (Konno et al., 2024). How Ano1 and Cftr work together in SW-type ionocytes in these teleosts will be an interesting avenue for future research. Such investigations are likely to reveal that the ionoregulatory machinery of the teleost SW-type ionocyte is more complex than is currently understood.

Our pharmacological studies further indicated the presence of a Cftr-independent mechanism for ionoregulation in the gills of SW-acclimated sea lamprey. DIDS is known to block many anion channels, including Clc chloride channel family members (such as Clcn2) and Ca^2+^-activated Cl^−^ channels (such as Ano1), but conspicuously does not affect Cftr (Harding et al., 2024). The insensitivity of Cftr to DIDS has been utilized to characterize the presence of Cftr in the teleost SW-type ionocyte (Marshall et al., 2002a). As the fish gill is a 3-dimensionally complex and fragile structure, electrophysiological studies targeting ion currents across sea lamprey gill tissue *ex vivo* are not feasible. Thus, we conducted a series of *in vivo* experiments that applied DIDS to the basolateral and apical surfaces of the gill epithelium using intraperitoneal and aqueous administration, respectively. Based on its limited membrane permeability (Cabantchik and Rothstein, 1972), when DIDS is applied aqueously it is unlikely to inhibit Ano1 in the basolateral membrane of ionocytes. Here, we found that aqueous but not intraperitoneal exposure to DIDS impaired osmoregulation in SW-exposed juvenile sea lamprey. Aqueous exposure to a Ca^+^-activated Cl^−^ channel inhibitor (CaCC-10) impaired osmoregulation in Japanese medaka, which also express *ano1* in the gills (Konno et al., 2024). Of course, the aqueous administration of DIDS does not strictly target the gill epithelium, and it is possible that DIDS inhibited Cl^−^ transport mechanisms in the intestinal epithelium, another prominent site for ion transport that directly interfaces with the external environment (via imbibed SW). The intestine of SW-acclimated sea lamprey is a site of net Cl^−^ absorption (Barany et al., 2020, 2021a), and thus inhibition of Cl^−^ transport by DIDS would presumably lead to reduced plasma Cl^−^ levels, not the increased levels we observed here. However, we cannot rule out an effect of DIDS on other tissues. Thus, we cautiously interpret our findings that aqueous exposure to DIDS had no effect in FW, but a robust effect to increase plasma Cl^−^ in SW, as additional evidence of a Cftr-independent Cl^−^ pathway in sea lamprey gills. Whether this pathway involves Ano1, which is highly abundant in these ionocytes, remains to be resolved. Future studies should further elucidate the localization and role of Ano1 in lamprey ionocytes while also considering whether other DIDS-sensitive Cl^−^ transporters may function in these ionocytes and contribute to ionoregulation in SW.

As extant members of the sister clade to all other vertebrates, lampreys and hagfishes are intriguing and useful models for generating hypotheses about vertebrate evolution. However, care should be taken when forming evolutionary hypotheses about vertebrate physiological processes based on comparative observations made from extant taxa, as there is no fossil record of physiological processes to test such hypotheses. With this in mind, we conservatively view our present description of a Cftr-independent salt-secretory ionocyte in sea lamprey gills as new information that will inform investigations attempting to resolve how ionoregulatory processes evolved in vertebrates.

One possible reason for the presence of Cftr-independent ionocytes in the sea lamprey gills could be that it constitutes the mechanism for salt secretion that was present in a common vertebrate ancestor. Once a matter of intense debate, recent analyses have established that early vertebrates evolved in near-shore subtidal and intertidal marine environments (Sallan et al., 2018). In members of the early branching vertebrate lineage Agnatha, represented by extant hagfishes and lampreys, gill Cftr does not appear to be involved in branchial ionoregulation in the marine environment. Recent genomic and transcriptomic analyses of the inshore hagfish (*Eptatretus burgeri*) showed that Cftr is undetectable in the genome of this species (Yamaguchi et al., 2024; Yu et al., 2024), and our results in the present study demonstrate that Cftr does not have a salt-secretory role in sea lamprey gills. Thus, it is possible that the absence of branchial Cftr function in agnathans partly reflects ionoregulatory mechanisms in the earliest vertebrates, which may have included salt secretion through a Cftr-independent mechanism still employed by extant anadromous lampreys. Alternatively, the presence of the Cftr-independent ionocyte in sea lamprey could reflect a species- or lamprey-specific adaptation acquired after the radiation of modern lampreys. Indeed, the radiation of crown-group lampreys may have been from a FW ancestor (Brownstein and Near, 2023). It is possible that when anadromy was acquired (or re-acquired) during the radiation of modern lampreys, a Cftr-independent mechanism for branchial Cl^−^ secretion was adopted by SW-type ionocytes. Further, the acquisition of a Cftr-independent mechanism for salt secretion may have occurred in bony fish taxa as well. Indeed, much of what is known about Cl^−^ secretory mechanisms in euryhaline and marine fishes is derived from a limited number of fishes, considering the many thousands of species within this category. Thus, Cftr-independent mechanisms for branchial Cl^−^ secretion may exist in euryhaline and marine fishes, which have simply not been examined yet.

The physiological function of Cftr in lamprey remains unknown. The overall primary sequence of lamprey Cftr is only 46% similar to human CFTR – much less than the ∼72% similarity between elasmobranch Cftr and human CFTR (Marshall and Singer, 2002; Marshall et al., 1991). The C-terminal region of human CFTR detected by the 24-1 antibody has a ∼66% similarity to the Cftr of teleosts (which it has been widely used to detect) and a ∼55% similarity to the lamprey Cftr. Our inability to detect Cftr in lamprey gills using this antibody aligns with the lack of transcriptional expression of *cftr* in lamprey gills but could also be due to a lower avidity of 24-1 for the lamprey Cftr. A closer inspection of the lamprey Cftr sequence structure revealed that it lacks consensus with human CFTR at critical phosphorylation sites (Cui et al., 2019). Importantly, lamprey Cftr contains a leucine at residue position 508 (residue number corresponds to human CFTR), whereas Cftr in gnathostomes contains a phenylalanine at this position (Bose et al., 2015). The residue Phe508 in human CFTR is functionally and clinically important. The vast majority of people with cystic fibrosis (CF) have a deletion of Phe508, which results in severe functional deficiencies in the CFTR protein (Rowe et al., 2005). Substitution of Leu for Phe at this position similarly reduces CFTR membrane trafficking (Thibodeau et al., 2005). Functional studies of lamprey Cftr *in vitro* showed that it differs considerably from human CFTR (Cui et al., 2019). Lamprey Cftr exhibits a reduced rate of activation by cAMP and responds poorly to small-molecule CFTR activators, potentiators and inhibitors (Cui et al., 2019). The structural and functional differences between the lamprey Cftr and the Cftrs from derived vertebrates raise the possibility that lamprey Cftr does not operate as a Cl^−^ channel at all, but rather as a transporter of some other anion, such as bicarbonate (Cui et al., 2019).

### Conclusion

Here, we present multiple lines of evidence that the mechanism for Cl^−^ secretion in the gills of sea lamprey is independent of Cftr, thus contrasting with the Cftr-dependent Cl^−^ secretory pathway of all other marine fishes examined to date. Although the origins of salt-secretory mechanisms in vertebrates may remain unresolved as a result of limitations in extant species available to study, the possibility that a novel molecular pathway for branchial Cl^−^ secretion exists in lampreys warrants further examination of the origins and evolution of salt-secretory physiology in vertebrates. Our work can form the basis for further molecular, functional and physiological studies on Cftr and Ano1 in basal vertebrates, including the protochordates, hagfishes, lampreys, elasmobranchs and chondrosteans to better understand how shifts in osmoregulatory and ionoregulatory physiology and the molecular evolution of Cftr supported vertebrate evolutionary transitions.

## Supplementary Material

10.1242/jexbio.250110_sup1 Supplementary information
