# Supplementary material for: A Cftr-independent, Ano1-rich seawater-adaptive ionocyte in sea lamprey gills
Source: J Exp Biol. 2025 Apr 2;228(7):jeb250110. doi: 10.1242/jeb.250110 (PMC11993260; doi:10.1242/jeb.250110)
Supplement: Supplementary information [file jexbio-228-250110-s1.pdf]

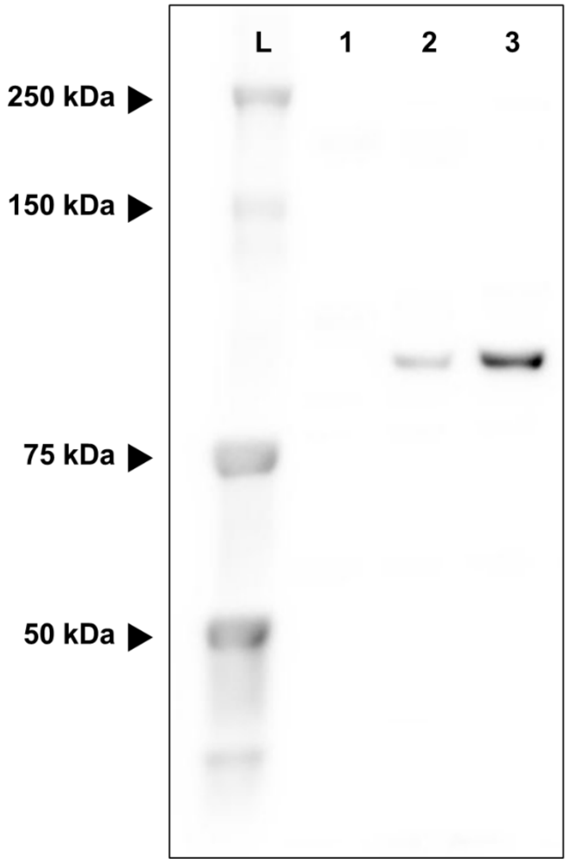

Fig. S1. Representative membrane from Western blot analysis. The complete lanes for chemiluminescent detection of sea lamprey Ano1 by the custom-made anti-sea lamprey Ano1 antibody are shown. Lane assignments are as follows: (1) FW-acclimated larvae, (2) FW-acclimated juvenile, (3) SW-acclimated juvenile. L, ladder.

Journal of Experimental Biology • Supplementary information

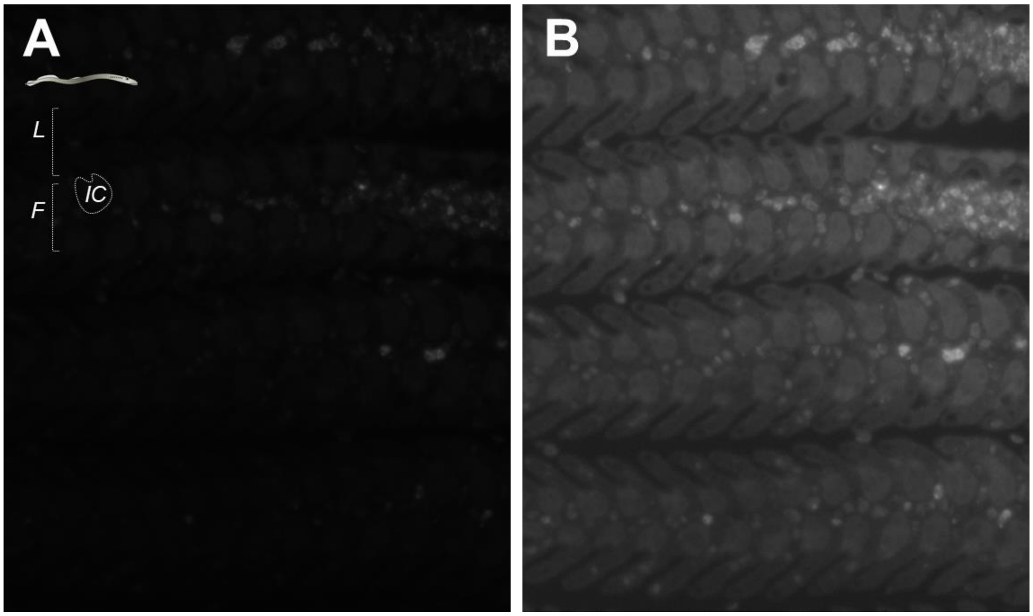

Fig. S2. Immunofluorescence microscopy image of secondary only control. The gills of SW-acclimated juvenile sea lamprey incubated with secondary antibody only. The two panels are the same image, with (**A**) depicting the standard exposure, and (**B**) depicting a longer exposure (enhanced brightness) for clarity of visual reference. Ionocyte clusters (*IC*) are located along the filament (*F*) between lamellae (*L*).

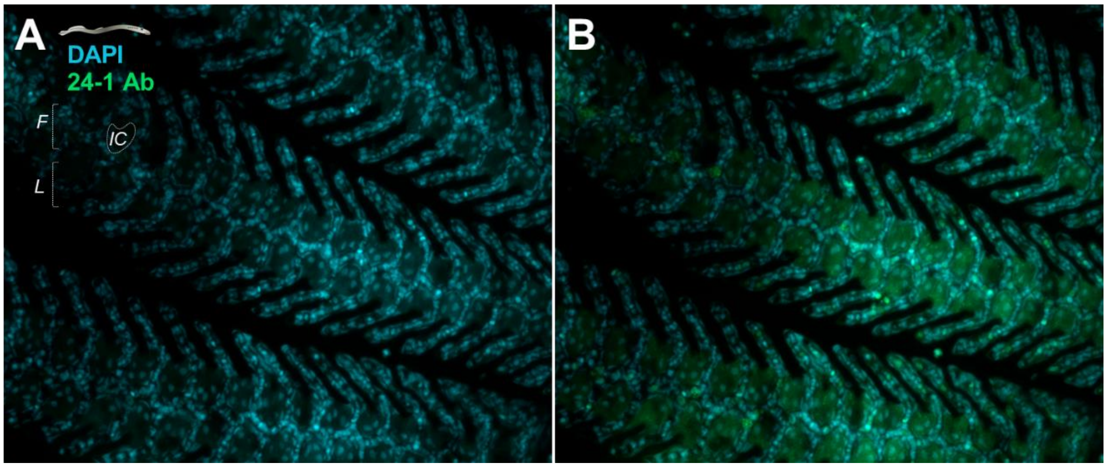

Fig. S3. Immunofluorescence microscopy image using the anti-Cftr antibody '24-1'. The gills of SW-acclimated juvenile sea lamprey incubated with the nuclear marker DAPI (blue) and the anti-Cftr antibody '24-1' (green). The two panels are the same image, with (**A**) depicting the standard exposure. A longer exposure (enhanced brightness) is depicted in (**B**) for visual clarity (no specific signal was detected). Ionocyte clusters (*IC*) are located along the filament (*F*) between lamellae (*L*).

Table S1. Raw data and differential expression analysis of an *a priori* and *a posteriori* selection of Cl^- or anion transporters in the branchial transcriptomes of larvae and juvenile (Juv) sea lamprey. Data available on Dryad: 10.5061/dryad.z34tmpgm3.

			Normalized Transcript Counts				DESeq2 Analysis			Wald Test Comparison			
**LOC#**	**GeneID**	**Gene Name**	**Larvae FPM**	**SE**	**Juv FPM**	**SE**	**baseMean**	**Log_2FC**	**SE**	**Statistic**	***P***	**P_{adj}**	**Sig?**
116950729	*nkcc*	*solute carrier family 12 member 2*	38.22	4.65	2393.49	266.73	20656.57	-6.17	0.23	-26.48	1.72E-154	9.65E-151	TRUE
116956436	*ano1*	*anoctamin 1*	18.76	1.25	52.89	4.32	565.91	-1.59	0.18	-9.03	1.69E-19	5.18E-18	TRUE
116950414	*clcn2*	*chloride channel protein 2-like*	1.11	0.13	6.2	0.9	51.17	-3.41	0.44	-7.74	1.04E-14	1.89E-13	TRUE
116949658	*ttyh2*	*protein tweety homolog 2-like*	8.02	0.84	17.17	1.9	193.05	-1.37	0.27	-5.16	2.48E-07	1.76E-06	TRUE
116951572	*ttyh1*	*protein tweety homolog 1-like*	5.28	0.6	11.38	1.31	138.23	-1.19	0.24	-5.01	5.60E-07	3.76E-06	TRUE
116949466	*slc26a6*	*solute carrier family 26 member 6-like*	19.33	1.16	17.85	0.68	13.61	-3.16	0.63	-5.01	5.59E-07	3.76E-06	TRUE
116942811	*lrrc8d*	*volume-regulated anion channel subunit LRRC8D-like*	5.58	0.65	11.14	0.75	116.85	-1.13	0.24	-4.71	2.46E-06	1.48E-05	TRUE
116943351	*clcc1*	*chloride channel CLIC like 1*	18.63	0.95	27.58	0.4	286.35	-0.66	0.15	-4.40	1.09E-05	5.84E-05	TRUE
116946045	*clcn7*	*chloride voltage-gated channel 7*	41.28	0.78	59.51	3.15	700.66	-0.56	0.14	-4.15	3.34E-05	1.63E-04	TRUE
116946971	*clcnkb*	*chloride channel protein ClC-Kb-like*	16.42	2.97	26.62	5.08	252.79	-1.48	0.41	-3.64	2.70E-04	1.08E-03	TRUE
116953150	*clic5*	*chloride intracellular channel protein 5-like*	18.6	0.84	28.74	2.03	234.75	-0.70	0.20	-3.55	3.82E-04	1.46E-03	TRUE
116936995	*lrrc8a*	*volume-regulated anion channel subunit LRRC8A-like*	8.34	0.3	11.91	0.75	70.60	-0.84	0.24	-3.48	4.94E-04	1.85E-03	TRUE
116951979	*bsnd*	*barttin CLCNK type accessory subunit beta*	10.91	1.01	20.54	2.68	68.71	-1.10	0.32	-3.40	6.76E-04	2.45E-03	TRUE
116938942	*lrrc8a*	*volume-regulated anion channel subunit LRRC8A-like*	45.48	1.69	57.92	1.82	792.41	-0.39	0.12	-3.25	1.17E-03	3.98E-03	TRUE
116951305	*slc26a6*	*solute carrier family 26 member 6-like*	49.65	2.37	55.76	3.18	320.66	-0.86	0.31	-2.83	4.73E-03	1.37E-02	TRUE
116958216	*lrrc8c*	*volume-regulated anion channel subunit LRRC8C-like*	6.95	0.52	8.57	0.55	93.42	-0.46	0.21	-2.17	2.98E-02	6.76E-02	FALSE
116947243	*pacc1*	*proton activated chloride channel 1*	17.12	0.35	20.84	1.53	296.39	-0.36	0.17	-2.13	3.34E-02	7.47E-02	FALSE
116938665	*ccc9*	*solute carrier family 12 member 8*	45.92	2.22	53.44	2.53	589.84	-0.31	0.15	-2.04	4.11E-02	8.90E-02	FALSE
116949685	*slc26a11*	*solute carrier family 26 member 11*	20.38	2.14	26.06	1.81	321.22	-0.33	0.20	-1.68	9.36E-02	1.75E-01	FALSE
116952042	*kcc2*	*solute carrier family 12 member 5-like*	0.86	0.25	1.48	0.37	11.32	-0.85	0.57	-1.50	1.35E-01	2.34E-01	FALSE
116944991	*clca1*	*calcium-activated chloride channel regulator 1-like*	6.68	0.45	6.39	0.59	58.38	-0.46	0.32	-1.42	1.56E-01	2.63E-01	FALSE
116937505	*slco2a1*	*solute carrier organic anion transporter family member 2A1*	50.26	6.88	30.4	2.24	34.99	-0.64	0.49	-1.31	1.91E-01	3.09E-01	FALSE
116955765	*best2*	*bestrophin-2-like*	52.94	3.86	56.02	3.87	800.05	-0.20	0.17	-1.20	2.32E-01	3.58E-01	FALSE
116957883	*ttyh1*	*protein tweety homolog 1-like*	15.62	0.17	20.12	0.8	11.58	-0.64	0.54	-1.17	2.41E-01	3.69E-01	FALSE
116937120	*lrrc8a*	*volume-regulated anion channel subunit LRRC8A-like*	2.07	0.32	2.49	0.27	22.44	-0.49	0.53	-0.92	3.59E-01	4.99E-01	FALSE
116946982	*ae2*	*anion exchange protein 2-like*	194.36	22.27	210.25	8.16	3419.32	-0.15	0.17	-0.89	3.75E-01	5.16E-01	FALSE
116948778	*ccc8*	*solute carrier family 12 member 9*	93.03	7.34	100.76	5.74	1598.34	-0.14	0.16	-0.86	3.90E-01	5.30E-01	FALSE
116946972	*clcnkb*	*chloride channel protein ClC-Kb-like*	9.4	0.8	9.9	0.57	61.24	-0.26	0.31	-0.84	4.01E-01	5.41E-01	FALSE
116954894	*clcn6*	*chloride transport protein 6-like*	6.78	0.85	7.07	0.54	41.06	-0.30	0.36	-0.84	4.03E-01	5.43E-01	FALSE
116937805	*nkcc*	*solute carrier family 12 member 2-like*	43.34	8.89	49.28	3.79	692.41	-0.22	0.28	-0.79	4.33E-01	5.72E-01	FALSE
116937010	*lrrc8c*	*volume-regulated anion channel subunit LRRC8C-like*	1.02	0.13	1.32	0.16	10.07	-0.29	0.56	-0.51	6.11E-01	7.27E-01	FALSE
116938813	*slc26a10*	*solute carrier family 26 member 10-like*	1.98	0.18	2.04	0.27	0.31	-1.63	3.49	-0.47	6.40E-01	NA	N/A
116949463	*pds*	*pendrin-like*	1.83	0.31	3.08	0.15	5.70	-0.22	0.78	-0.28	7.79E-01	8.55E-01	FALSE
116944961	*clic5*	*chloride intracellular channel protein 5-like*	72.23	13.52	72.68	4.27	1098.44	-0.04	0.27	-0.14	8.88E-01	9.32E-01	FALSE
116945081	*clca1*	*calcium-activated chloride channel regulator 1-like*	0.07	0.04	0.11	0.03	0.56	-0.36	2.58	-0.14	8.89E-01	NA	N/A
116951316	*cftr*	*CF transmembrane conductance regulator*	3.1	0.42	3.53	0.15	0.56	-0.33	2.58	-0.13	8.98E-01	NA	N/A
116944545	*ano1*	*anoctamin-1-like*	1.14	0.15	1.51	0.06	5.53	-0.09	0.84	-0.10	9.19E-01	9.51E-01	FALSE
116946215	*ttyh1a*	*protein tweety homolog 1-A-like*	129.1	6.25	124.06	2.66	1969.35	0.02	0.10	0.25	8.01E-01	8.71E-01	FALSE
116937300	*lrrc8e*	*volume-regulated anion channel subunit LRRC8E-like*	0.39	0.01	0.39	0.04	0.11	1.01	3.53	0.29	7.76E-01	NA	N/A
116954669	*ae2*	*anion exchange protein 2-like*	6.19	1.1	10.33	2.36	67.90	0.11	0.35	0.31	7.55E-01	8.36E-01	FALSE
116945083	*clca1*	*calcium-activated chloride channel regulator 1-like*	3.85	0.29	4.06	0.12	0.61	0.86	2.53	0.34	7.35E-01	NA	N/A
116955745	*best2*	*bestrophin-2-like*	9.82	0.54	11.03	1.01	65.02	0.12	0.24	0.50	6.16E-01	7.31E-01	FALSE
116939820	*ano1*	*anoctamin-1-like*	3.58	0.42	3.45	0.36	0.77	1.34	2.12	0.63	5.27E-01	NA	N/A
116957008	*kcc2*	*solute carrier family 12 member 5-like*	4.52	0.21	4.88	0.26	3.31	0.77	0.94	0.81	4.17E-01	5.57E-01	FALSE
116954190	*kcc4*	*solute carrier family 12 member 7*	39.14	1.99	34.95	2.64	532.23	0.15	0.17	0.87	3.85E-01	5.25E-01	FALSE
116938201	*ano6*	*anoctamin-6-like*	5.05	0.92	2.8	0.38	1.49	1.69	1.79	0.94	3.46E-01	4.87E-01	FALSE
116957515	*clcn3*	*chloride voltage-gated channel 3*	33.81	3.81	31.4	1.59	338.22	0.18	0.16	1.12	2.65E-01	3.98E-01	FALSE
116945082	*clca1*	*calcium-activated chloride channel regulator 1-like*	0.27	0.12	0.17	0.04	1.48	2.47	1.86	1.33	1.84E-01	3.00E-01	FALSE
116955412	*clic5*	*chloride intracellular channel protein 5-like*	79.45	6.24	62.73	3.39	835.93	0.39	0.14	2.80	5.19E-03	1.49E-02	TRUE
116953033	*clic5*	*chloride intracellular channel protein 5-like*	14.01	2.33	8.12	1.22	153.45	0.97	0.35	2.81	4.96E-03	1.43E-02	TRUE
116944987	*clca1*	*calcium-activated chloride channel regulator 1-like*	3.48	0.63	1.94	0.09	23.38	2.15	0.46	4.64	3.52E-06	2.05E-05	TRUE
116940953	*ndcbe*	*sodium-driven chloride bicarbonate exchanger-like*	74.33	4.69	29.93	1.5	708.64	1.35	0.14	9.64	5.27E-22	2.06E-20	TRUE
116957041	*clccc*	*chloride channel protein C-like*	34.36	3.88	11.11	0.44	181.37	2.67	0.27	9.95	2.42E-23	1.07E-21	TRUE

Journal of Experimental Biology • Supplementary information
